# Learning curve analysis of single-port thoracoscopic combined subsegmental resections

**DOI:** 10.3389/fonc.2023.1072697

**Published:** 2023-02-09

**Authors:** Yizhou Huang, Maohui Chen, Shuliang Zhang, Taidui Zeng, Guanglei Huang, Bin Zheng, Chun Chen

**Affiliations:** ^1^ Key Laboratory of Cardio-Thoracic Surgery, Fujian Medical University, Fujian Province University, Fuzhou, China; ^2^ Department of Thoracic Surgery, Fujian Medical University Union Hospital, Fuzhou, China; ^3^ National Key Clinical Specialty of Thoracic Surgery, Fuzhou, China

**Keywords:** subsegmental resection, learning curve, three-dimensional reconstruction and simulation, combined dimensionality reduction method, video-assisted thoracoscopy

## Abstract

**Background:**

Combined subsegmental surgery (CSS) is considered to be a safe and effective resection modality for early-stage lung cancer. However, there is a lack of a clear definition of the technical difficulty classification of this surgical case, as well as a lack of reported analyzes of the learning curve of this technically demanding surgical approach.

**Methods:**

We performed a retrospective study of single-port thoracoscopic CSS performed by the same surgeon between April 2016 and September 2019. The combined subsegmental resections were divided into simple and complex groups according to the difference in the number of arteries or bronchi which need to be dissected. The operative time, bleeding and complications were analyzed in both groups. Learning curves were obtained using the cumulative sum (CUSUM) method and divided into different phases to assess changes in the surgical characteristics of the entire case cohort at each phase.

**Results:**

The study included 149 cases, including 79 in the simple group and 70 in the complex group. The median operative time in the two groups was 179 min (IQR, 159-209) and 235 min (IQR, 219-247) p < 0.001, respectively. And the median postoperative drainage was 435 mL (IQR, 279-573) and 476 mL (IQR, 330-750), respectively, with significant differences in postoperative extubation time and postoperative length of stay. According to the CUSUM analysis, the learning curve for the simple group was divided by the inflection point into 3 phases: Phase I, learning phase (1st to 13th operation); Phase II, consolidation phase (14th to 27th operation), and Phase III, experience phase (28th to 79th operation), with differences in operative time, intraoperative bleeding, and length of hospital stay in each phase. The curve inflection points of the learning curve for the complex group were located in the 17th and 44th cases, with significant differences in operative time and postoperative drainage between the stages.

**Conclusion:**

The technical difficulties of the simple group of single-port thoracoscopic CSS could be overcome after 27 cases, while the technical ability of the complex group of CSS to ensure feasible perioperative outcomes was achieved after 44 operations.

## Background

With the implementation of lung cancer screening programs using computed tomography (CT) and low-dose CT (LDCT) in high-risk patients, an increasing number of small early-stage lung cancers (≤2 cm) are being detected (1). Many studies have shown that sublobar resection produces the same oncological outcomes as lobectomy in patients with stage I non-small cell lung cancer (2). While wedge resection has been reported as a risk factor for local recurrence and poorer survival (3, 4), segmental resection or subsegmental resection benefits from its removal of venous and lymphatic drainage in the intersegmental plane, providing acceptable surgical outcomes (5, 6). Anatomical segmental resection is increasingly proposed as an alternative to lobectomy for small-sized lesions, particularly those presenting with ground glass opacity (GGO) (7). However, a large proportion of small-sized peripheral ground-glass shaded nodules in clinical practice are not located in the center of the lung segments, but between them, and it is difficult to meet their marginal requirements with segmental resection alone. Combined segmentectomy or lobectomy can remove these nodules, but too much normal lung tissue is excised. While combined subsegmental surgery (CSS) can preserve lung function as much as possible while ensuring tumor margins.

The CSS is usually considered more technically demanding than segmental lung resection, because of the variety of vessels and bronchi that need to be dealt with, generally in larger numbers and at a more dissected distance from the hilum. Therefore, CSS requires thorough preoperative reconstruction and surgical planning to ensure the safe performance of multiple subsegmental resections. Related studies have shown that thoracoscopic CSS with 3-dimensional (3D) navigation is a safe technique for intersegmental nodal resection, saving more lung parenchyma and ensuring safe margins for anatomical resection (8, 9). It was also shown that FEV1 in each lobe after CSS was higher than that after multisegmental resection (0.3 ± 0.2 vs. 0.2 ± 0.2 l, p=0.07), which is effective for maintaining lung function in each lobe (10). The CSS learning curve study reported by Zhang et al. showed that in single-port thoracoscopic subsegmental resection, a surgical procedure of 28 cases was required to achieve a level of surgical proficiency (11), but fewer cases of complex subsegmental resection were included, while the selected cases were not stratified for difficulty. Regarding the criteria for classifying simple and complex lung segment resections, scholars have proposed classifying them according to the type of intersegmental plane designed, i.e. whether they are complex segmental resections according to linear intersegmental planes or non-linear complex intersegmental planes (12, 13). On this basis, we believe that the combined subsegmental resection technique is characterized by a complex and variable intersegmental plane and can therefore be further classified for technical difficulty based on the number of intraoperative off-segmental target lung tissue vessels and bronchi.

In this study, the learning curve for CSS was investigated using cumulative sum (CUSUM) analysis to assess the surgical characteristics and postoperative outcomes of patients undergoing simple and complex combined subsegmental lung resection, and to analyze the pattern of the learning curve comparing simple and complex combined subsegmental lung resection, which can be used to guide the safe performance of subsequent procedures.

## Method

### Patients

The study population covered 149 patients who underwent CSS by the same surgeon at Fujian Medical University’s Union Hospital between April 2016 and September 2019. Patients who received single-port thoracoscopic CSS for less than or equal to 2 cm GGO were included in the study and divided into simple and complex groups according to the difference in the number of arteries or bronchi which need to be dissected. A simple CSS is defined as a procedure in which the number of vessels dissected and the number of bronchi were both less than or equal to 3. In contrast, if one of the number of vessels or bronchi removed is greater than 3, it was considered complex CSS. Patients found to have intraoperative thoracic adhesions were excluded. Information was collected on age, gender, site of resection, duration of surgery, intraoperative bleeding, final pathological diagnosis, duration of chest tube placement, length of hospital stay, intraoperative and postoperative complications. Learning curves were constructed to analyze the differences in operative time and intraoperative and postoperative complications between periods in the consecutive surgical cohorts. The study was approved by the review committee of the Union Hospital of Fujian Medical University. The data are anonymous, and the requirement for informed consent was therefore waived.

### Surgical procedure

The surgical approach is determined by the lesion characteristics on the preoperative CT scan of the chest. The extent of surgical resection and the final surgical plan are based on the size of the nodule and the adjacent structures of the lesion, with the principle of ensuring resection of the tumor margins and maximum preservation of lung function. The appropriate margin for resection should be greater than or equal to 2 cm or greater than or equal to the diameter of the lesion. Preoperatively, all patients are reconstructed in three dimensions using the IQQA-3D system (EDDA technology), using thin-section enhanced CT as the data source. In this system, the lung areas are planned and accurately reconstructed according to the tracheal branches and the trachea, arteries and veins of the lung lobes. The location and extent of the lung nodules are marked, the lung area is delineated and a resection margin sphere is created at 2cm from the lesion margin or greater than the tumor diameter. The reconstruction is analyzed to observe the relationship of the resection margin sphere to the bronchi and lung tissue, and the extent of resection is determined first, and then the target lung segment vessels to be resected are determined accordingly. In each case, an experienced surgeon discusses and formulates the resection plan and discusses its feasibility, assessing the structure of the target lung segment and the sequence of treatment.

After general anesthesia, the patient is operated with the assistance of a single-port thoracoscope. A 3.5-4.0 cm incision was made in the fourth rib space in the mid-axillary line. The target arteries and bronchi were isolated to reveal them in the sequence planned preoperatively, ligated and then dissected with an ultrasonic knife. Both lungs are then inflated with 100% oxygen and the target lung tissue is atrophied by ventilating one lung for 15 min. For the management the inter-segmental plane, a “combined dimensional reduction method” (14) is used, whereby the subsegmental plane is treated according to the guidance of the intersegmental distension-atrophy divide, first separating the inter-segmental plane from the hilum distally with the ultrasonic knife, stretching the target lung segment to one side and meticulously separating nearly three-quarters of the proximal parenchyma so that the remaining unsegmented target parenchyma is sufficiently thin and lies in a two-dimensional plane. This allows the anastomosis to be quickly positioned in the resection plane to cut through the remaining parenchyma. Following sampling of the mediastinal lymph nodes, a lung leak test was performed. The bronchial stump was examined for significant air leaks and blood leakage, and hemostatic material was placed on the surgical wound.

### Statistical analysis

SPSS Statistics 26.0 (IBM Corporation, Armonk, NY) was used for all statistical analyzes. Continuous variables were compared using t-tests or Wilcoxon rank sum tests. Categorical data were compared using the chi-square test. Differences in variables between the two groups were considered statistically significant at the p<0.05 level. In this study, the cumulative sum method was used to analyze the learning curve. Cumulative sums were used to analyze the duration of surgery for a series of consecutive operations to see if the operation was proficient and if the learning curve was overcome.

## Results

**Figure 1:**
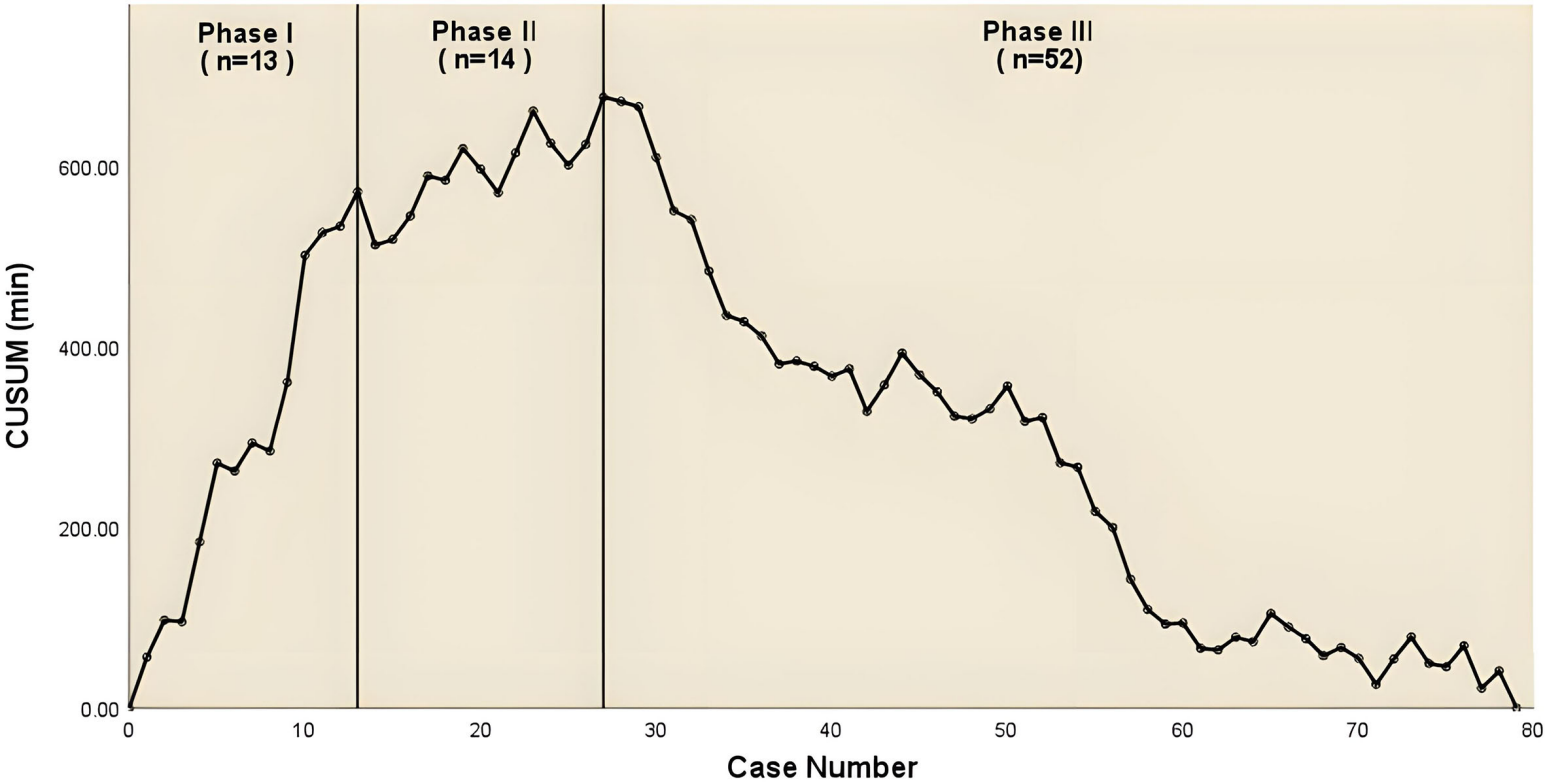
The CUSUM chat for operative time of simple combined subsegmental resecion.

**Figure 2:**
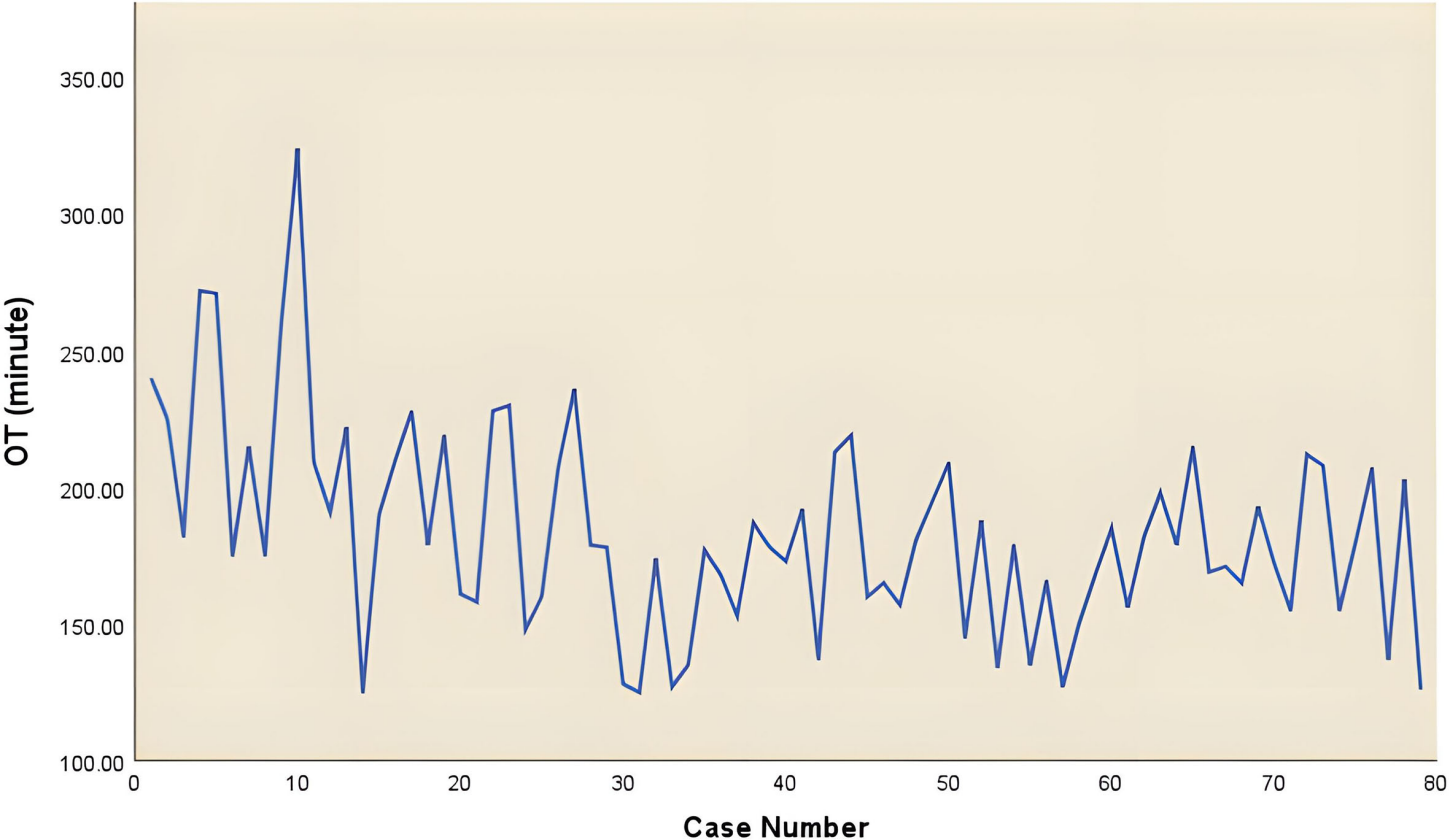
The operative time of simple combined subsegmental resecion.

One hundred and forty-nine consecutive patients underwent combined single-port thoracoscopic subsegmental resection, 79 in the simple group and 70 in the complex group. The median age in the two groups was 49 years (IQR, 39-57), 54 years (IQR, 45-60) p<0.05, median operative time was 179 min (IQR, 159-209), 235 min (IQR, 219-247) p<0.001, median postoperative drainage 435 ml (IQR, 279-573), 476 ml (IQR, 330-750) p<0.05, median postoperative extubation time 4 days (IQR, 3-4), 4 days (IQR, 3-5) p<0.05, median postoperative hospital stay 4 days (IQR, 3-4), 4 days (IQR, 3-6) p<0.05, and postoperative lung infection rates of 13.9% and 18.6%, respectively. **Table 1** shows the baseline characteristics and other perioperative data for all cases. According to the CUSUM analysis, cut-off points were established in the curve area due to increasing and decreasing operative times, and the CUSUM OT for the simple group. **Figure 1** suggests that the learning curve for the simple group was divided by inflection points into 3 phases: phase I, learning phase (1st to 13th operation); phase II, consolidation phase (14th to 27th operation), and phase III, experience phase (28th to 79th operation), with each The median operative time and intraoperative bleeding in each stage were 222 min (IQR, 191-260), 199 min (IQR, 160-226), 173 min (IQR, 155-187), 50 ml (IQR, 50-50), 30 ml (IQR, 30-50), 30 ml (IQR, 20-50), with statistically significant differences. **Table 2** showed the basal characteristics and other perioperative data for cases in the simple group at each stage. **Figure 2** demonstrated the operative time of simple CSS.

**Table 1:** Comparisons of patient characteristics and operative parameters in simple and complex groups.

Characteristics	Simple group (n= 79)	Complex group (n= 70)	(P value)
Sex, n (%)			
Male	60 (75.9)	49 (70.0)	0.415
Age			
Median (IQR), y	49 (39-57)	54 (45-60)	0.020
ASA score			
Median (IQR)	2 (2-2)	2 (2-2)	0.929
History of hypertension, n (%)			0.103
Yes	14	6	
History of diabetes, n (%)			0.596
Yes	4	5	
History of cigarette smoking, n (%))			0.309
Yes	8	11	
History of alcohol consumption, n (%)			0.474
Yes	16	11	
Location, n (%)			0.074
RUL	37	19	
RML	0	0	
RLL	8	15	
LUL	31	33	
LLL	3	3	
Tumor size, cm			<0.001
0 to ≤ 1	67	46	
1 to ≤ 2	11	23	
>2	1	1	
operative time			<0.001
Mean (SD), min	179 (159-209)	235 (219-247)	
Bleeding			0.370
Median (IQR), mL	50(20-50)	50(30-50)	
Drainage			
Median (IQR), d	4(3-4)	4(3-5)	0.002
Median (IQR), mL	435 (279-573)	476 (330-750)	0.028
Length of hospital stay			
Median (IQR), POD	4(3-4)	4(3-6)	0.004
Postoperative pulmonary infection, n (%)			0.443
Yes	11(13.9)	13(18.6)	
Pathologic diagnosis, n (%)			0.192
Minimally invasive	67	52	
Invasive adenocarcinoma	9	16	
Benign	3	2	

IQR, Interquartile range, ASA, American Society of Anesthesiologists; RUL, right upper lobe; RML, right middle lobe; RLL, right lower lobe; LUL, left upper lobe; LLL, left lower lobe; OT, operative time; SD, standard deviation; POD, postoperative day.

**Table 2:** Interphase comparisons of patient characteristics and operative parameters in all simple cases.

Characteristics	Phase I (n= 13)	Phase II (n= 14)	Phase III (n=52)	Phase I vs Phase II	Phase I & II vs Phase III	(P value)
Sex, n (%)						
Male	10 (76.9)	10 (71.4)	40 (76.9)	0.749	0.780	0.910
Age						
Median (IQR), y	41 (36-54)	52 (47-58)	49 (39-56)	0.120	0.605	0.201
ASA score				0.217	0.897	0.450
Median (IQR)	2 (1-2)	2 (2-2)	2 (2-2)			
History of hypertension, n (%)				0.692	0.894	0.912
Yes	2	3	9			
History of diabetes, n (%)				0.134	0.496	0.154
Yes	2	0	2			
History of cigarette smoking, n (%))				0.957	0.566	0.847
Yes	1	1	6			
History of alcohol consumption, n (%)				0.937	0.389	0.688
Yes	2	2	12			
Location, n (%)				0.228	0.416	0.405
RUL	9	5	23			
RML	0	0	0			
RLL	0	4	4			
LUL	4	5	22			
LLL	0	0	3			
Tumor size, cm				0.980	0.975	0.957
0 to ≤ 1	12	14	41			
1 to ≤ 2	0	0	11			
>2	1	0	0			
operative time				0.048	<0.001	<0.001
Mean (SD), min	222 (191-260)	199 (160-226)	173 (155-187)			
Bleeding				0.028	0.099	0.044
Median (IQR), mL	50 (50-50)	30 (30-50)	30 (20-50)			
Drainage						
Median (IQR), d	4 (3-4)	4 (4-5)	3(3-4)	0.080	0.025	0.020
Median (IQR), mL	445 (287-570)	459 (366-588)	425 (249-556)	0.771	0.264	0.496
Length of hospital stay						
Median (IQR), POD	4 (4-4)	4 (4-5)	4 (3-4)	0.325	0.015	0.033
Postoperative pulmonary infection, n (%)				0.937	0.870	0.983
Yes	2 (15.4)	2 (14.3)	7 (13.5)			
Pathologic diagnosis, n (%)				0.307	0.947	0.590
Minimally invasive	12	11	44			
Invasive adenocarcinoma	1	2	6			
Benign	0	1	2			

IQR, Interquartile range, ASA, American Society of Anesthesiologists; RUL, right upper lobe; RML, right middle lobe; RLL, right lower lobe; LUL, left upper lobe; LLL, left lower lobe; OT, operative time; SD, standard deviation; POD, postoperative day.

**Figure 3:**
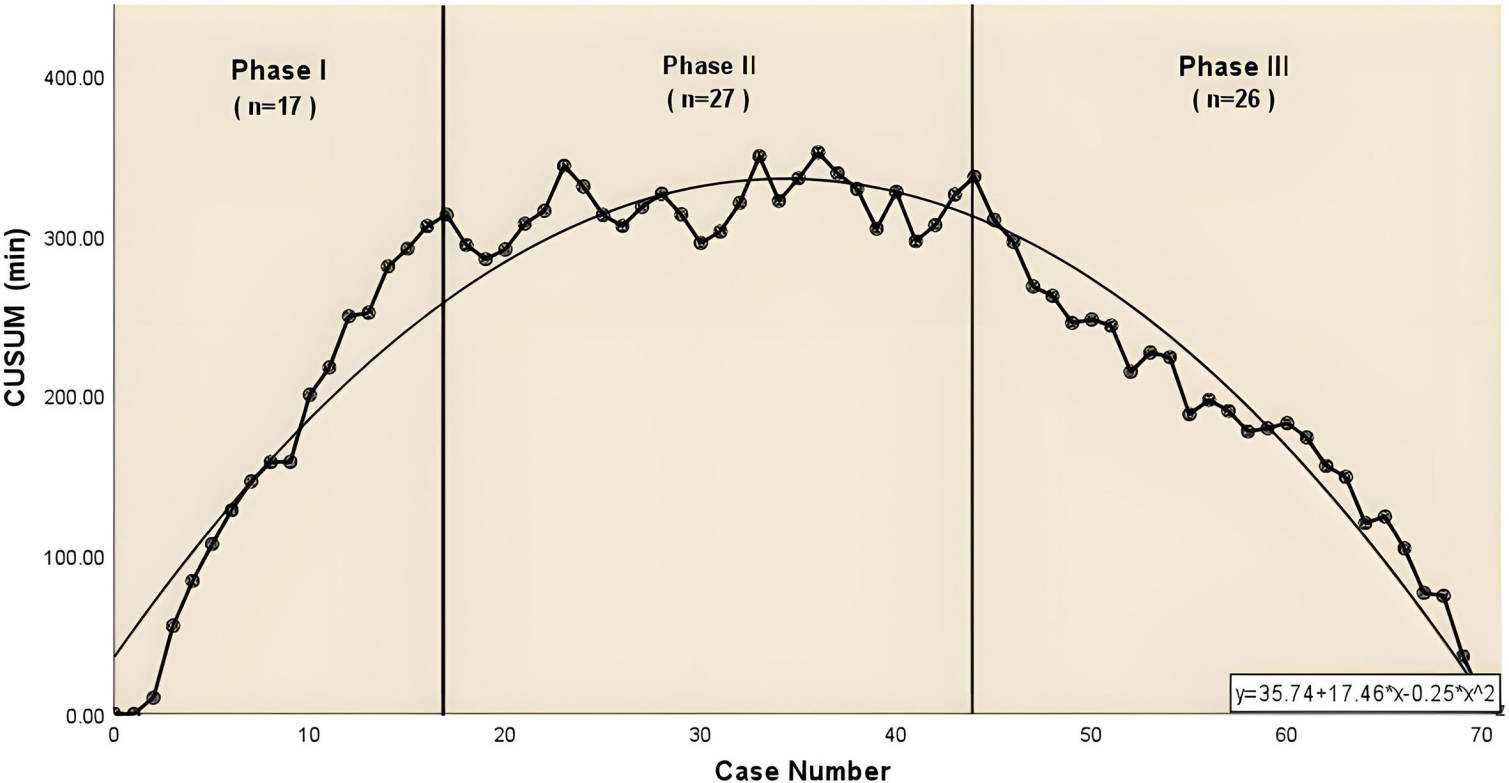
The CUSUM chat for operative time of complex combined subsegmental resecion. The dashed line represents the curve of best fit for the plot (a second-order polynomial with equation CUSUM_OT_= −0.25×case number ^2^ + 17.46×case number +35.74.

**Figure 4:**
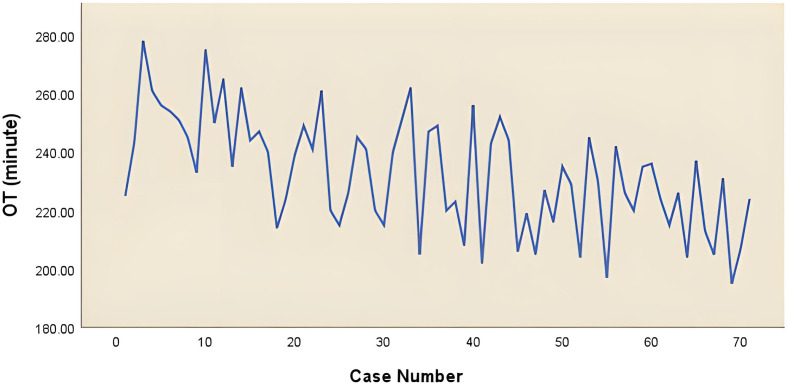
The operative time of complex combined subsegmental resecion.

For the complex group (**Figure 3**), the curve inflection points of the learning curve for the complex group are located in the 17th and 44th cases, and we can distinguish 3 phases in the figure: phase 1, the learning phase (1st to 17th operation) suggests a longer than median operative time; phase 2, the consolidation phase (18th to 44th operation) remains dynamically stable and suggests an approximately equal to the median operation time. ; phase 3, the experience phase (45th to 70th operation) suggests a less than median operative time. The median operative time and median postoperative drainage in each phase were 250 min (IQR, 243-261), 240 min (IQR, 220-248), 222 min (IQR, 206-230), 855 ml (IQR, 360-1010), 500 ml (IQR, 369-738), 460 ml (IQR, 210-665). There were no significant differences between the stages of surgical bleeding, postoperative extubation time, postoperative hospital stay and postoperative lung infection rates. **Table 3** showed the baseline characteristics and other perioperative data for cases in the complex group at each stage. **Figure 4** illustrated the operative time of simple CSS.

**Table 3:** Interphase comparisons of patient characteristics and operative parameters in all complex cases.

Characteristics	Phase 1 (n= 17)	Phase 2 (n= 27)	Phase 3 (n=26)	(P value)
Sex, n (%)				
Male	11 (64.7)	16 (59.3)	22 (84.6)	0.077
Age				
Median (IQR), y	57 (47-62)	57 (46-60)	50 (43-58)	0.409
ASA score				
Median (IQR)	2 (2-2)	2 (2-2)	2 (2-2)	0.410
History of hypertension, n (%)				0.501
Yes	2	3	1	
History of diabetes, n (%)				0.498
Yes	1	1	3	
History of cigarette smoking, n (%))				0.066
Yes	3	7	1	
History of alcohol consumption, n (%)				0.681
Yes	3	5	3	
Location, n (%)				0.338
RUL	7	5	7	
RML	0	0	0	
RLL	1	8	6	
LUL	9	13	11	
LLL	0	1	2	
Tumor size, cm				0.668
0 to ≤ 1	11	16	19	
1 to ≤ 2	6	11	6	
>2	0	0	1	
operative time				<0.001
Mean (SD), min	250 (243-261)	240 (220-248)	222 (206-230)	
Bleeding				0.835
Median (IQR), mL	50(30-50)	50 (20-50)	30 (30-50)	
Drainage				
Median (IQR), d	5 (3-7)	4 (3-5)	3 (4-5)	0.151
Median (IQR), mL	855 (360-1010)	500 (369-738)	460 (210-665)	0.004
Length of hospital stay				0.080
Median (IQR), POD	5 (3-7)	4 (4-6)	4 (3-5)	
Postoperative pulmonary infection, n (%)				0.094
Yes	5 (29.4)	6 (22.2)	2 (7.7)	
Pathologic diagnosis, n (%)				0.829
Minimally invasive	13	19	20	
Invasive adenocarcinoma	4	8	4	
Benign	0	0	2	

IQR, Interquartile range, ASA, American Society of Anesthesiologists; RUL, right upper lobe; RML, right middle lobe; RLL, right lower lobe; LUL, left upper lobe; LLL, left lower lobe; OT, operative time; SD, standard deviation; POD, postoperative day.

## Discussion

JCOG0802 and 0804 studies have shown that lung segments are preferable to lobes in early stage lung cancer (15). However, some nodules are not centrally located in the lung segment and are not suitable for segmental lung resection. Some studies have shown that CSS is safe and feasible for such nodules. For GGOs located between segments, CSS removes venous and lymphatic drainage in the intersegmental plane, and adjacent subsegmental resection rather than a larger wedge resection provides a safe margin (16, 17). In addition, CSS reduces the degree of lung volume reduction and is therefore considered more minimally invasive than segmental resection for smaller nodules, preserving lung function in each lobe by avoiding lobectomy or multiple segmental resections (18). The preservation of lung function associated with fewer resections may be particularly important in those patients with borderline lung function and in those who will require additional lung resections in the future to treat multiple lung cancer. The primary objective of this study was to analyze the learning curve pattern of CSS and to guide the safe operation of subsequent surgeries.

The surgical difficulty of CSS varies considerably from one individual to another, in two aspects: firstly, the intersegmental plane of CSS is usually irregular and varies widely; secondly, the number of vessels and airways to be dissected is variable. As the intersegmental plane of CSS is variable and difficult to quantify, we have therefore considered the number of vessels or tracheas to be treated as a criterion for simple or complex CSS, based on clinical experience. A simple CSS is defined as a procedure in which the number of vessels dissected and the number of bronchi are both less than or equal to 3, such as RS2b+S3a resection, LS1+2 (a+b) resection, etc. In contrast, if one of the number of vessels or bronchi removed is greater than 3, it is considered complex CSS, such as RS6b+S8ai+S9a resection, LS1+2(a+b) +S3c resection, etc. Compared to simple CSS, complex CSS requires the surgeon to identify segmental arteries and veins in greater detail, especially to differentiate between the numerous intersegmental and intra-segmental veins, to separate and divide appropriate bronchi more peripherally, and to identify and manage more complex intersegmental planes. To our best knowledge, this is the first study of CSS learning curves stratified by surgical difficulty. Furthermore, our study is the first study to present an attempt to differentiate between Simple CSS and Difficult CSS.

The learning curve is a graphical representation of the temporal relationship between the surgeon’s mastery of a given task and the amount of time spent performing the case. Cumulative sums (CUSUM) can help to visually identify trends in a data set and have proved particularly valuable when analyzing learning curves (19, 20). In the series presented in this study joint subsegmental resections were divided into simple and complex groups, where 27 cases were required in the simple group to become proficient in simple joint subsegmental resections and 32 cases were required in the complex group to gain technical proficiency in the application of complex subsegmental resections. Both in the simple and complex groups, the initial learning period showed a longer operative time, but intraoperative bleeding and postoperative complications were in a more acceptable range, which can be attributed to the correct preoperative 3D reconstruction and planning of the surgical procedure. Variations in vascular and bronchial structures may increase operative time and the risk of accidental bronchial injury, but with recent advances such as image processing and artificial intelligence 3D reconstruction allowing proof of the precise structure of the pulmonary arteries and veins, this allows surgeons to perform CSS more safely and effectively (21–23).

A physician with extensive experience in segmental resection can control the operative time and perioperative complications more quickly during the accumulation of CSS experience. Our results show that the learning curve for simple CSS requires a learning process of 27 cases before the experience phase can be entered, and the experience phase requires a cumulative experience process of 52 cases. We found no significant difference in intraoperative bleeding or postoperative drainage between the learning process (first 27 cases) and the experience-building process (second 52 cases). In the previous report, the number of cases for the learning curve of pulmonary segment surgery was 33, which is similar to the results of our study (24). We believe this is because simple CSS is similar in difficulty to segmental lung resection and requires similar numbers of vessels and bronchi to be dissected, so with prior experience in segmental lung surgery and preoperative 3D reconstruction, simple CSS can be mastered with only a smaller number of cases.

The application of a preoperative 3D reconstruction system for identification of lung segment structures and surgical planning can help to overcome the learning curve of complex CSS more smoothly. The learning curve for the complex group of combined subsegmental resections was divided into three phases, namely learning, plateau and experience, with 17 and 44 cases as the inflection points. In all cases, stage 1 represents the initial part of the learning curve and includes 17 cases. Meanwhile the Stage 2 plateau phase includes 27 cases, which means that once the initial phase of the learning curve has passed, more experience is gained and subsequently the experience phase is entered. The complex and diverse anatomy makes complex combined subsegmental resections technically more difficult. Our team, with the aid of the IQQA-3D system, identifies the segmental structures and locates the nodes while showing the 3D relationships between segmental bronchi, arteries and veins. The target subsegments were identified based on a 2cm marginal sphere constructed around the nodes, ensuring safe margins in surgical planning. Our research team has demonstrated in previous studies that IQQA can detect most arterial segmental, venous and bronchial variation in surgical planning, with a variable frequency of 61.6% and 17.8% for segmental arteries and veins respectively (25). We therefore believe that the use of 3D images for surgical simulation and intraoperative one-to-one correspondence between the actual anatomy and the virtual anatomy, enabling real-time navigation during the procedure, can reduce the difficulty of the technique on subsegmental or sub-subsegmental resection and improve the accuracy of the procedure.

There are a number of factors that can affect the learning curve of CSS. For example, pleural adhesions can have a significant impact on operative time, so in this study, we excluded patients with dense pleural adhesions. In patients with incomplete lung fissures this makes the procedure more difficult, but we have sufficient experience in single-port thoracoscopic surgery that there are no substantial difficulties at the technical level. The limitations of this study are its retrospective nature and the fact that it was performed in a single study center. Postoperative survival benefits of simple and complex CSS require long-term follow-up.

## Conclusion

In summary, single-port thoracoscopic CSS is a safe and feasible for small lung lesions, with perioperative data on intraoperative bleeding and postoperative complications in the acceptable range. The technical difficulties in the simple group could be overcome after 27 of these cases, while the technical ability to ensure feasible perioperative outcomes with combined subsegmental resection in the complex group was achieved after 44 procedures.

## Data availability statement

The raw data supporting the conclusions of this article will be made available by the authors, without undue reservation.

## Ethics statement

The study was approved by the review committee of the Union Hospital of Fujian Medical University. The data are anonymous, and the requirement for informed consent was therefore waived (Lines 90 to 91 of the manuscript).

## Author contributions

YH, MC, and CC contributed to conception and design of the study. BZ organized the database. MC performed the statistical analysis. YH wrote the first draft of the manuscript. SZ, TZ, GH, and BZ wrote sections of the manuscript. All authors contributed to manuscript revision, read, and approved the submitted version.
